# Supplementary material for: Update on Interventional Management of Neuropathic Pain: A Delphi Consensus of the Spanish Pain Society Neuropathic Pain Task Force
Source: Medicina (Kaunas). 2022 Apr 30;58(5):627. doi: 10.3390/medicina58050627 (PMC9146461; doi:10.3390/medicina58050627)
Supplement: Supplementary file 1 [file medicina-58-00627-s001.zip › example of original questionnaire in Spanish.pdf]

NOMBRE:
APELLIDO 1:
APELLIDO 2:
CARGO EN EL CENTRO DE TRABAJO:

SECCIÓN: TOXINA BOTULÍNICA

1. La toxina botulínica (TB) tiene evidencias de eficacia y seguridad y a pesar de ser un uso compasivo figura como tercera línea en muchas de las guías de DN

VALORE	0 totalmente en desacuerdo	1 en desacuerdo	2 indiferente	3 de acuerdo	4 totalmente de acuerdo

2. Se recomienda su utilización en pacientes refractarios añadida al tratamiento estándar

VALORE	0 totalmente en desacuerdo	1 en desacuerdo	2 indiferente	3 de acuerdo	4 totalmente de acuerdo

3. Desconocemos el NNT, que por el pequeño tamaño de los estudios podría ser muy bajo (menor de 2), aun que algunos estudios más grandes con buena metodología hablan de NNT superiores a 7.

VALORE	0 totalmente en desacuerdo	1 en desacuerdo	2 indiferente	3 de acuerdo	4 totalmente de acuerdo

4. La TB es un tratamiento útil y eficaz en ancianos con DN refractario

VALORE	0 totalmente en desacuerdo	1 en desacuerdo	2 indiferente	3 de acuerdo	4 totalmente de acuerdo

5. La utilidad de la TB parece ser menor en pacientes con hipoestesia y alteración de la sensibilidad térmica, por lo que en principio no serán candidatos a ensayo terapéutico con la misma

VALORE	0 totalmente en desacuerdo	1 en desacuerdo	2 indiferente	3 de acuerdo	4 totalmente de acuerdo

6. Las dosis analgésicas pueden variar desde pocas unidades hasta 200, dependiendo principalmente del tamaño de la zona de dolor

VALORE	0 totalmente en desacuerdo	1 en desacuerdo	2 indiferente	3 de acuerdo	4 totalmente de acuerdo

7. Existe una evidencia muy débil (series de casos) de utilización de la TB en zonas perineurales, pero no podemos descartar su utilidad en base a las publicaciones existentes. Por ello se recomienda hacer estudios con buena metodología para poder demostrar la seguridad y la eficacia de esta nueva y prometedora modalidad de tratamiento con TB

VALORE	0 totalmente en desacuerdo	1 en desacuerdo	2 indiferente	3 de acuerdo	4 totalmente de acuerdo

SECCIÓN: INFILTRACIONES Y BLOQUEOS

8. Los bloqueos nerviosos o las infiltraciones disponen de un nivel de evidencia 2, grado B de recomendación, para el tratamiento de la NPH

VALORE	0 totalmente en desacuerdo	1 en desacuerdo	2 indiferente	3 de acuerdo	4 totalmente de acuerdo

9. En la NPH, en el 1º nivel de tratamiento tenemos las inyecciones subcutáneas de toxina botulínica / corticoides; o el bloqueo del ganglio estrellado (herpes facial sin

NPH), y en 2º nivel el bloqueo paravertebral o epidural y los bloqueos nerviosos con esteroides (para alivio de síntomas)

VALORE	0 totalmente en desacuerdo	1 en desacuerdo	2 indiferente	3 de acuerdo	4 totalmente de acuerdo

10. Se recomienda no utilizar bloqueos simpáticos para lesiones de NPH

VALORE	0 totalmente en desacuerdo	1 en desacuerdo	2 indiferente	3 de acuerdo	4 totalmente de acuerdo

11. Los corticoides perineurales reducen el dolor neuropático

VALORE	0 totalmente en desacuerdo	1 en desacuerdo	2 indiferente	3 de acuerdo	4 totalmente de acuerdo

12. En el SDRC los bloqueos simpáticos con AL no son efectivos

VALORE	0 totalmente en desacuerdo	1 en desacuerdo	2 indiferente	3 de acuerdo	4 totalmente de acuerdo

13. Los bloqueos ilieoinguinal e ileohipogástrico combinando anestésico local con corticoide pueden ser efectivos para tratar el dolor crónico postquirúrgico tras cirugía de hernia

VALORE	0 totalmente en desacuerdo	1 en desacuerdo	2 indiferente	3 de acuerdo	4 totalmente de acuerdo

SECCIÓN: RADIOFRECUENCIA

14. La radiofrecuencia pulsada (RFP) puede considerarse como una alternativa eficaz para el tratamiento de la neuralgia postherpética (NPH)

VALORE	0 totalmente en desacuerdo	1 en desacuerdo	2 indiferente	3 de acuerdo	4 totalmente de acuerdo

15. En el tratamiento de la NPH se deberían considerar terapias de alta frecuencia y larga duración

VALORE	0 totalmente en desacuerdo	1 en desacuerdo	2 indiferente	3 de acuerdo	4 totalmente de acuerdo

16. La aplicación de RFP precóz reduce el dolor del herpes y la incidencia de NPH

VALORE	0 totalmente en desacuerdo	1 en desacuerdo	2 indiferente	3 de acuerdo	4 totalmente de acuerdo

17. La radiofrecuencia convencional (RFC) es más efectiva que la RFP para la neuralgia del trigémino idiopática

VALORE	0 totalmente en desacuerdo	1 en desacuerdo	2 indiferente	3 de acuerdo	4 totalmente de acuerdo

18. Los resultados para la RF térmica del ganglio de Gasser o en sus ramas periféricas tiene resultados similares

VALORE	0 totalmente en desacuerdo	1 en desacuerdo	2 indiferente	3 de acuerdo	4 totalmente de acuerdo

19. En el SDRC no se puede recomendar la radiofrecuencia

VALORE	0 totalmente en desacuerdo	1 en desacuerdo	2 indiferente	3 de acuerdo	4 totalmente de acuerdo

SECCIÓN: NEUROMODULACIÓN

20. La terapia de estimulación de cordones posteriores (SCS) está indicada en síndrome de cirugía de espalda fallida (FBSS) y en síndrome de dolor regional complejo (SDRC)

VALORE	0 totalmente en desacuerdo	1 en desacuerdo	2 indiferente	3 de acuerdo	4 totalmente de acuerdo

21. En FBSS es tan eficaz la estimulación de alta frecuencia (HF10) como la de baja frecuencia

VALORE	0 totalmente en desacuerdo	1 en desacuerdo	2 indiferente	3 de acuerdo	4 totalmente de acuerdo

22. Se debe considerar la estimulación del ganglio de la raíz dorsal (GRD) si se trata de dolor radicular

VALORE	0 totalmente en desacuerdo	1 en desacuerdo	2 indiferente	3 de acuerdo	4 totalmente de acuerdo

23. En dolor neuropático localizado estaría indicada la estimulación el GRD

VALORE	0 totalmente en desacuerdo	1 en desacuerdo	2 indiferente	3 de acuerdo	4 totalmente de acuerdo

24. Podemos utilizar estimulación nerviosa periférica en dolor neuropático periférico postquirúrgico o posttraumático

VALORE	0 totalmente en desacuerdo	1 en desacuerdo	2 indiferente	3 de acuerdo	4 totalmente de acuerdo

25. En dolor neuropático crónico, la terapia PENS (percutaneous electrical nerve stimulation) tiene utilidad

VALORE	0 totalmente en desacuerdo	1 en desacuerdo	2 indiferente	3 de acuerdo	4 totalmente de acuerdo

26. La estimulación BURST puede ser tan eficaz como la estimulación tónica para el tratamiento del dolor neuropático.

VALORE	0 totalmente en desacuerdo	1 en desacuerdo	2 indiferente	3 de acuerdo	4 totalmente de acuerdo

SECCIÓN: ULTIMAS PREGUNTAS

GENERO:

EDAD:

PROVINCIA DE EJERCICIO:

TITULARIDAD DE CENTRO DE TRABAJO:

AÑOS DE EXPERIENCIA PROFESIONAL (EXCLUYENDO FORMACIÓN):
