# Supplementary material for: Update on Interventional Management of Neuropathic Pain: A Delphi Consensus of the Spanish Pain Society Neuropathic Pain Task Force
Source: Medicina (Kaunas). 2022 Apr 30;58(5):627. doi: 10.3390/medicina58050627 (PMC9146461; doi:10.3390/medicina58050627)
Supplement: Supplementary file 1 [file medicina-58-00627-s001.zip › examples of emails.pdf]

Consenso en Dolor Neuropático (1)

De GT Neuropático SED
Destinatario secretaria@sedar.es [redacted]
Cco
Fecha

Estimado/a panelista.

Ante todo pedirte disculpas por el retraso en el envío de este correo.

Deberíamos haber realizado el envío el viernes pasado. Pero nos hemos dado cuenta que tanto el trabajo de leer bibliografía, como el de plantear las preguntas y responder al Delphi es demasiado grande.

Así que, tareas administrativas aparte (como la de crear este correo electrónico y buscar a aquellos que no han facilitado el suyo el día de la Jornada), hemos decidido dividir el trabajo en dos secciones:
1) sección farmacología
2) sección intervencionismo (en aras del trabajo práctico, hemos incluido la toxina botulínica en esta sección).

Además, vamos a aumentar los plazos de lectura de la bibliografía y de respuesta de las preguntas.

Recogeremos los datos de aquellos que participen según las respuestas dadas en el Delphi. Como dijimos, todos los que participen estarán en la publicación como grupo agregado. Podéis elegir participar en uno indistintamente o en ambos.

En este correo de hoy os enviamos dos enlaces para descargar la bibliografía recomendada:
Descarga la bibliografía sobre Farmacología
Descarga la bibliografía sobre Intervencionismo
(los enlaces estarán disponibles sólo una semana)

Dentro de unos días volveremos a enviar otro correo. En él tendréis para descargar los resúmenes de las ponencias y un enlace para responder a la primera tanda de preguntas Delphi.

Una vez más, disculpad el retraso y ánimo con la lectura,
Saludos cordiales del Grupo de Trabajo Dolor Neuropático.

webmail.splink.es

SED | Sociedad Española del Dolor 💗 — Roundcube Webmail :: 1ª ronda Delphi intervencionismo.

Acerca de — gtneuropatico@sedolor.es — Cerrar sesión

roundcube — Correo — Contactos — Configuración

Volver | Redactar | Responder | Responder ... | Reenviar | Eliminar | Mover | Imprimir | SPAM | Marcar | Más

- Entrada
- Borradores
- **Enviados**
- SPAM
- Papelera

1ª ronda Delphi intervencionismo.

Mensaje 272 de 286

De: GT Neuropático SED

Destinatario: gtneuropatico@sedolor.es

Cco: [redacted]

Fecha: 2020-03-03 12:57

Buenas tardes.

Hoy empezamos la primera ronda del Delphi de intervencionismo.
Recuerda que aquí, por temas organizativos, incluimos la toxina botulínica.

Al igual que el Delphi de farmacología, tienes toda la bibliografía relevante enviada hace meses.
En este enlace puedes descargar los resúmenes de los temas

Y, al igual que el Delphi de farmacología, daremos 1 mes de plazo para contestar a las preguntas de la primera ronda.
La primera ronda de intervencionismo se cerrará a finales de marzo.
Haz click aquí para responder a las preguntas sobre intervencionismo.

[redacted]

Saludos cordiales,
--
Ancor Serrano Afonso
Coordinador Grupo Trabajo Dolor Neuropático SED

webmail.splink.es

SED | Sociedad Española del Dolor 💗

Roundcube Webmail :: Vuelta a la normalidad. Nuevamente Delphi.

Acerca de

gtneuropatico@sedolor.es Cerrar sesión

roundcube

Correo Contactos Configuración

Volver Redactar Responder Responder ... Reenviar Eliminar Mover Imprimir SPAM Marcar Más

- Entrada
- Borradores
- **Enviados**
- SPAM
- Papelera

Vuelta a la normalidad. Nuevamente Delphi.

Mensaje 269 de 286

De GT Neuropático SED

[redacted]

Cco

Fecha 2020-05-21 10:17

Buenos días a todas y a todos.

Espero que estés todos bien.
La crisis sanitaria del Covid nos ha cogido a todos desprevenidos y a medias de varias tareas.
En cuanto al GTDN, estábamos en mitad de los Delphi. Faltaba cerrar el Delphi de farmacología, quedaba una semana para cerrarlo, y estábamos a mitad del Delphi de intervencionismo. Todo ello en la primera ronda de preguntas.

Una vez el brote epidémico parece que ha ido bajando, hemos vuelto a la normalidad.
Por eso creo indicado retomar el Delphi de nuevo.

Empezaremos por dónde lo dejamos el lunes 22 de mayo. Enviaré un mail con el enlace del Delphi de farmacología (será la última semana) y el enlace de intervencionismo (estaremos por la mitad).

Intentaré hacer un esfuerzo para reenviar la bibliografía a aquellos que lo necesiten.

Saludos cordiales,

--
Ancor Serrano Afonso
Coordinador Grupo Trabajo Dolor Neuropático SED

webmail.splink.es

SED | Sociedad Española del Dolor 💗 | Roundcube Webmail :: Inicio de SEGUNDA RONDA del Delphi INTERVENCIONISMO

Acerca de | gtneuropatico@sedolor.es | Cerrar sesión

roundcube | Correo | Contactos | Configuración

Volver | Redactar | Responder | Responder ... | Reenviar | Eliminar | Mover | Imprimir | SPAM | Marcar | Más

- Entrada
- Borradores
- **Enviados**
- SPAM
- Papelera

Inicio de SEGUNDA RONDA del Delphi INTERVENCIONISMO

Mensaje 244 de 286

De: GT Neuropático SED
Destinatario: gtneuropatico@sedolor.es
Cco: [redacted]
Fecha: 2020-09-03 13:39

Buenas tardes.

Espero que tú y tu entorno estén bien en este tiempo de pandemia.

Una vez acabado el periodo estival, estamos analizando los resultados del primer Delphi.
Al recibir este correo significa que estás en la segunda ronda.

EN BREVE enviaremos un correo con el enlace para rellenar el cuestionario para aquellas preguntas en las que no ha habido consenso en la primera ronda.

Al finalizar, enviaremos otro corro con otro enlace para que indiques tus preferencias de tratamiento.
Este segundo enlace es algo más farragoso y explicaremos bien cómo entrar y cómo rellenar. Se requiere la colaboración de cada uno para poder tener un consenso gráfico.

Saludos cordiales y ánimo.

--
Ancor Serrano Afonso
Coordinador Grupo Trabajo Dolor Neuropático SED

webmail.splink.es

SED | Sociedad Española del Dolor 💗 | Roundcube Webmail :: 2º ronda Delphi INTERVENCIONISMO

Acerca de | gtneuropatico@sedolor.es | Cerrar sesión

roundcube | Correo | Contactos | Configuración

Volver | Redactar | Responder | Responder ... | Reenviar | Eliminar | Mover | Imprimir | SPAM | Marcar | Más

- Entrada
- Borradores
- **Enviados**
- SPAM
- Papelera

2º ronda Delphi INTERVENCIONISMO

Mensaje 162 de 286

De: GT Neuropático SED

Destinatario: [redacted]

Fecha:

[redacted].xlsx (...

Hola [redacted].

Te envío una tabla de excel con tus resultados, la media y mediana del conjunto para que valores si quieres continuar o cambiar tu puntuación a cada pregunta.

Abre la tabla y sigue las instrucciones.

Si no pudieses abrir el enlace, **DESPUÉS** de comparar tus resultados, abre el siguiente enlace y rellena la encuesta. Gracias.

Pincha aquí para rellenar la 2ª encuesta Delphi

--

Ancor Serrano Afonso

Coordinador Grupo Trabajo Dolor Neuropático SED

First mail.

Title: Consensus in Neuropathic Pain.

First of all, I apologize for the delay in sending this email.

We should have shipped last Friday. But we have
realized that both the work of reading bibliography, as well as that of
posing the questions and answering the Delphi is too big.

So, aside from administrative tasks (such as creating this email
email and look for those who have not provided theirs on the day
of the Conference), we have decided to divide the work into two sections:
1) pharmacology section
2) interventionism section (for the sake of practical work, we have
including botulinum toxin in this section).

In addition, we are going to increase the deadlines for reading the bibliography and
answer of the questions.

We will collect the data of those who participate according to the answers
given in Delphi. As we said, everyone who participates will be in
the post as a group added. You can choose to participate in one
indistinctly or both.

In this email today we send you two links to download the
recommended bibliography:
Download the bibliography on Pharmacology [1]
Download the bibliography on Interventionism [2]
(links will be available for one week only)

In a few days we will send another email. In it you will have
to download the abstracts of the presentations and a link to
answer the first set of Delphi questions.

Once again, sorry for the delay and cheer up with the reading,
Best regards from the Neuropathic Pain Working Group.

--
Ancor Serrano Afonso
SED Neuropathic Pain Working Group Coordinator

Second mail.

Title: 1st round Delphi interventionism.

Good afternoon.

Today we start the first round of the Delphi interventionism.
Remember that here, for organizational reasons, we include botulinum toxin.

Like the Delphi of pharmacology, you have all the relevant bibliography sent months ago.
In this link you can download the summaries of the topics

And, like the Pharmacology Delphi, we will give 1 month to answer the questions in the first round.
The first round of interventionism will close at the end of March.
Click here to answer the questions about interventionism.

Best regards,
--
Ancor Serrano Afonso
SED Neuropathic Pain Working Group Coordinator

Third mail.

Title: Back to normal. Delphi again.

Good morning to each and everyone.

I hope you are all well.
The Covid health crisis has caught us all off guard and in the middle of various tasks.
Regarding the GTDN, we were in the middle of the Delphi. The pharmacology Delphi remained to be closed, there was a week left to close it, and we were halfway through the interventionism Delphi. All this in the first round of questions.

Once the epidemic outbreak seems to have subsided, we have returned to normal.
That's why I think it's advisable to go back to Delphi again.

We will pick up where we left off on Monday May 22nd. I will send an email with the Delphi pharmacology link (it will be the last week) and the interventionism link (we will be in the middle).

I will try to make an effort to forward the bibliography to those who need it.

Best regards,

--
Ancor Serrano Afonso
SED Neuropathic Pain Working Group Coordinator

Fourth mail

Title: Start of the SECOND ROUND of Delphi INTERVENTIONISM

Good afternoon.

I hope you and your surroundings are well in this time of pandemic.

Once the summer period is over, we are analyzing the results of the first Delphi.
Receiving this email means that you are in the second round.

SHORTLY we will send an email with the link to fill out the questionnaire for those questions in which there was no consensus in the first round.

At the end, we will send another mail with another link for you to indicate your treatment preferences.
This second link is somewhat more cumbersome and we will explain how to enter and how to fill it out. The collaboration of each one is required in order to have a graphic consensus.

Kind regards and encouragement.

--
Ancor Serrano Afonso
SED Neuropathic Pain Working Group Coordinator

Last mail.

Title: 2nd round Delphi INTERVENTIONISM

Hello XXXXX.

I send you an excel table with your results, the mean and median of the set so that you can decide if you want to continue or change your score for each question.

Open the table and follow the instructions.

If you cannot open the link, AFTER comparing your results, open the following link and fill out the survey. Thank you.

Click here to fill out the 2nd Delphi survey

--
Ancor Serrano Afonso
SED Neuropathic Pain Working Group Coordinator
