# Supplementary material for: Update on Interventional Management of Neuropathic Pain: A Delphi Consensus of the Spanish Pain Society Neuropathic Pain Task Force
Source: Medicina (Kaunas). 2022 Apr 30;58(5):627. doi: 10.3390/medicina58050627 (PMC9146461; doi:10.3390/medicina58050627)
Supplement: Supplementary file 1 [file medicina-58-00627-s001.zip › FIIDOLNE2 v1-1 Supp Digital Content.pdf]

Supplemental Digital Content

Supplemental Digital Content 1.doc

Supplemental Digital Content 2.doc

Supplemental digital content #1: Supplementary Figure S1: Box plots of the distribution of responses in round 1.

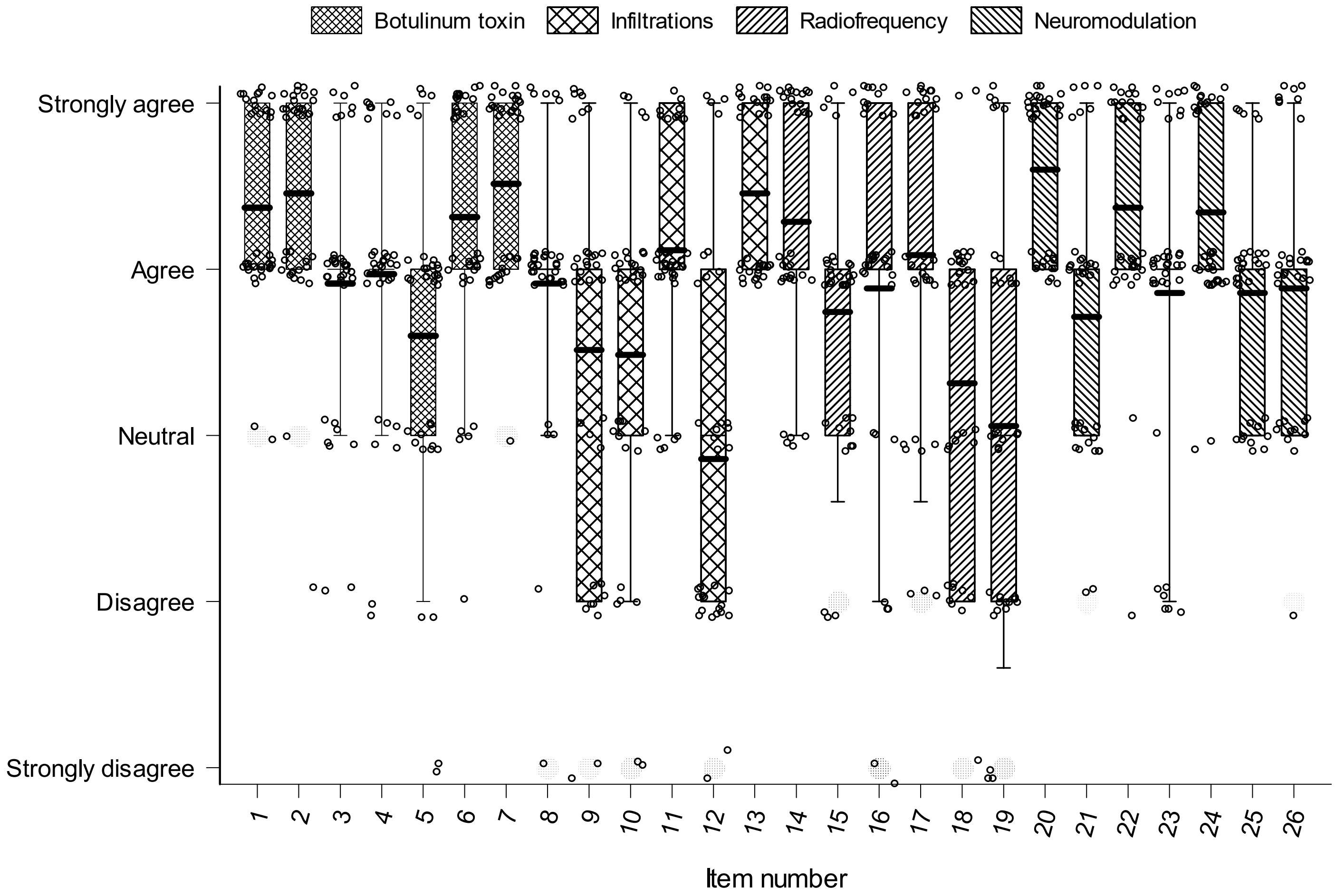

The boxes depict the 1st and 3rd quartiles (25th and 75th percentiles) and the whiskers the 10th and 90th percentiles, the thick horizontal lines are the means of the item responses, and the small circles represent each panelist's individual responses with some jitter to depict them separately. The boxes of some items are absent because they are collapsed over the median (when the 25th, 50th and 75th percentiles overlap, generally at value 3 "Agree").

Supplemental digital content #2: Supplementary Figure S2: Box plots of the distribution of responses in round 2.

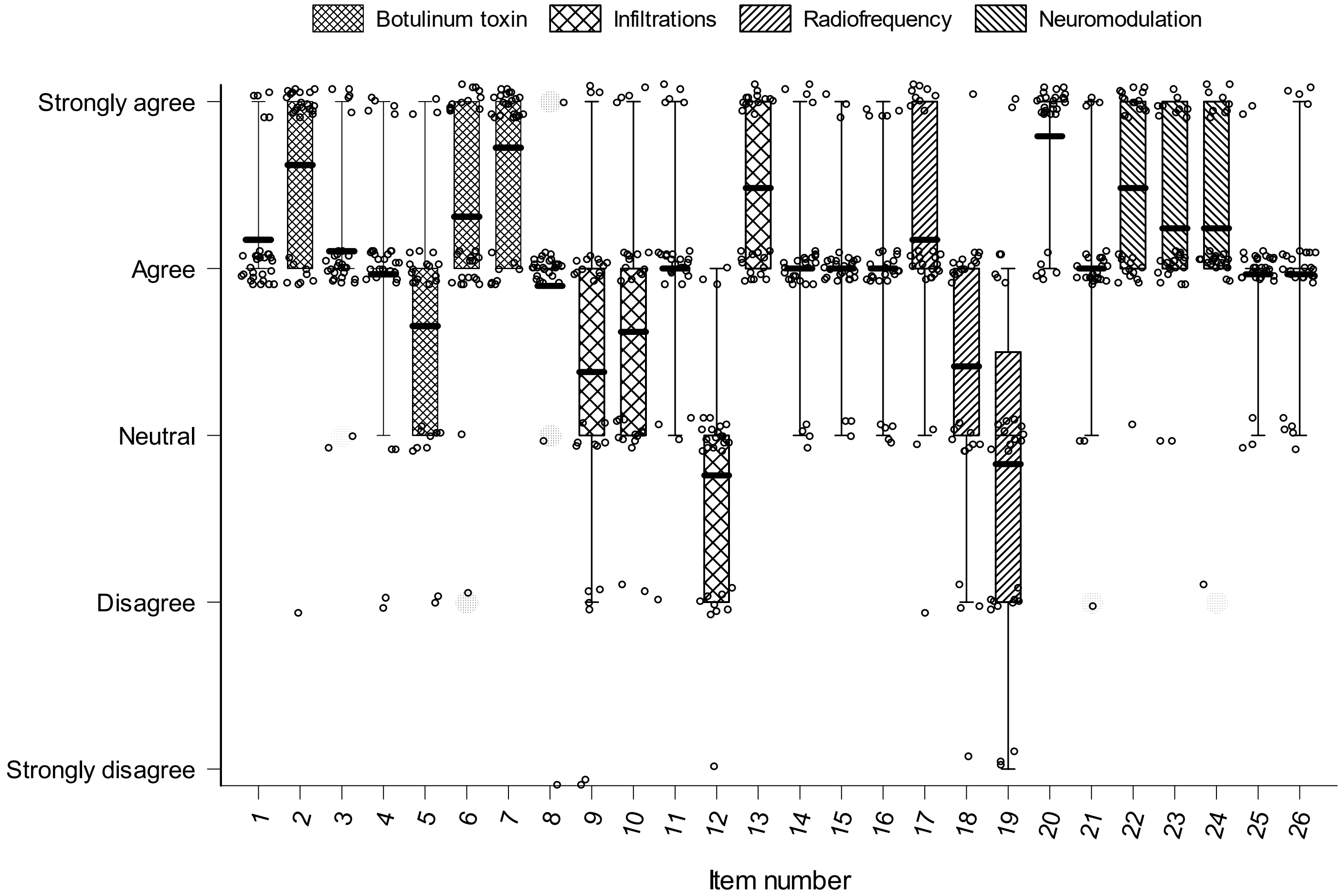

The boxes depict the 1st and 3rd quartiles (25th and 75th percentiles) and the whiskers the 10th and 90th percentiles, the thick horizontal lines are the means of the item responses, and the small circles represent each panelist's individual responses with some jitter to depict them separately. There are fewer boxes than in the figure of round 1 because many of them are collapsed over the median (when the 25th, 50th and 75th percentiles overlap, generally at value 3 "Agree").
